# The integration of head and body cues during the perception of social interactions

**DOI:** 10.1177/17470218231181001

**Published:** 2023-06-22

**Authors:** Elin H Williams, Bhismadev Chakrabarti

**Affiliations:** 1 Centre for Autism, School of Psychology and Clinical Language Sciences, University of Reading, Reading, UK; 2 India Autism Centre, Kolkata, India; 3 Department of Psychology, Ashoka University, Sonipat, India

**Keywords:** Social interaction, perception, cue integration, autism

## Abstract

Humans spend a large proportion of time participating in social interactions. The ability to accurately detect and respond to human interactions is vital for social functioning, from early childhood through to older adulthood. This detection ability arguably relies on integrating sensory information from the interactants. Within the visual modality, directional information from a person’s eyes, head, and body are integrated to inform where another person is looking and who they are interacting with. To date, social cue integration research has focused largely on the perception of isolated individuals. Across two experiments, we investigated whether observers integrate body information with head information when determining whether two people are interacting, and manipulated frame of reference (one of the interactants facing observer vs. facing away from observer) and the eye-region visibility of the interactant. Results demonstrate that individuals integrate information from the body with head information when perceiving dyadic interactions, and that integration is influenced by the frame of reference and visibility of the eye-region. Interestingly, self-reported autistics traits were associated with a stronger influence of body information on interaction perception, but only when the eye-region was visible. This study investigated the recognition of dyadic interactions using whole-body stimuli while manipulating eye visibility and frame of reference, and provides crucial insights into social cue integration, as well as how autistic traits affect cue integration, during perception of social interactions.

## Introduction

Humans are a profoundly social species and routinely process rich social information in their daily lives. The ability to quickly and accurately perceive individual agents, as well as the interactions and nature of relationships between individuals, is crucial for the successful navigation of our social world. We are quick to identify whether two people who are standing in close proximity to one another are engaged in a social interaction or behaving independently. While research has made significant progress in elucidating the nature of perception of individual agents, research has only recently started to investigate the processes underlying visual recognition of social interactions.

Interestingly, recent research shows that dyads positioned to imply an interaction are recognised more quickly and accurately than dyads facing away from each other (Papeo et al., 2017, 2019; Vestner et al., 2019). This search advantage for interacting dyads is suggested to be due to the strong directional cues (e.g., face, nose, and feet) present within these arrangements (Vestner et al., 2020). In addition, interacting individuals are processed in different regions of cortex compared with non-interacting individuals (Abassi & Papeo, 2020; Isik et al., 2017; Walbrin et al., 2018). These recent findings suggest that individuals positioned to imply an interaction are not perceived as two isolated individuals, but as two interacting individuals, and should thus be investigated as such.

In face-to-face social interactions, interacting individuals continuously exchange social signals such as facial expressions, body gestures, speech, and gaze. Gaze has a dual-function (Cañigueral & Hamilton, 2019); it tells us where our interaction partner is looking (Frischen et al., 2007) and what they might be thinking (Baron-Cohen et al., 1997) while also relaying the same information about our gaze behaviour to them. Thus, the ability to accurately judge the direction of another’s gaze is crucial in understanding complex and dynamic social environments such as social interactions. Unsurprisingly, humans exhibit a high degree of accuracy in judging the gaze direction of others (e.g., Bock et al., 2008; Gibson & Pick, 1963; Symons et al., 2004), and the human eye is suggested to have evolved to promote this ability (Kobayashi & Kohshima, 1997, 2001).

Although perceiving the direction of another’s gaze is crucial in accurately estimating the focus of their attention, accurate gaze estimation requires the integration of various other informative cues in our environments such as directional information from another person’s head (Balsdon & Clifford, 2017; Wollaston, 1824) and body (Moors et al., 2015). However, although the primary need for integration of social cues is during social situations that typically involve more than one person, social cue integration research has focused mostly on the visual perception of single individuals. In addition, the extent to which body information is integrated with head and eye-region information during gaze perception has been investigated to a limited extent.

**Figure 1.**
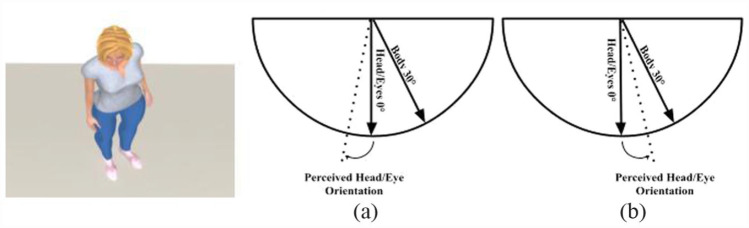
An illustration of the (a) overshoot and (b) towing effects (adapted from Moors et al., 2016).

Observers quickly and accurately judge the direction of gaze when directional cues of the eyes and head of isolated individuals are aligned (Langton, 2000; Ricciardelli & Driver, 2008; Seyama & Nagayama, 2005). However, when the eyes and the orientation of the head are misaligned, the integration of these cues introduces biases. For example, when the eyes of a looker are pointing directly towards an observer but the head is turned laterally, perceived gaze direction shifts in the direction opposite the head. This has been termed the overshoot, or repulsive, effect (Langton et al., 2000). This bias may be caused by a change in the amount of visible white sclera on either side of the iris when a person’s eyes are fixated while the head rotates, in a similar way to when gaze is averted but the head remains pointing forward (Anstis et al., 1969; Otsuka et al., 2014). To counteract this overshoot effect caused by a change in eye-region information, the towing, or attractive, effect (Maruyama & Endo, 1983) attempts to reduce the error in perceived gaze direction by utilising head information as a direct cue, pulling perceived gaze direction back towards the veridical (Otsuka et al., 2014). The overshoot effect has also been observed for the perception of head orientation in the presence of a misaligned body cue (Moors et al., 2015; Figure 1).

Social cue integration has been shown to vary across different contexts. Participants integrate and weight sensory evidence differently depending on the type of judgement they are making about the gaze of another person (Balsdon & Clifford, 2018). When participants judge the relative direction of another’s gaze (i.e., allocentric perspective), a stronger overshoot effect of the head is observed compared with when observers judge whether or not gaze is directed at them (i.e., egocentric perspective).

In addition, the integration of social cues during gaze perception is influenced by individual differences. For example, individuals with schizophrenia, who show impairments in self-referential gaze perception (Hooker & Park, 2005; Rosse et al., 1994; Tso et al., 2012), show no differences in gaze estimation accuracy when judging whether gaze is directed to the left, right, or straight ahead (i.e., making a judgement about the relative direction of gaze; Seymour et al., 2017). Thus, individuals with schizophrenia show differences while judging the direction of gaze in relation to themselves (i.e., egocentric judgement), while they show no such differences when judging the relative direction of gaze (i.e., allocentric judgement). Enhanced self-referential perception of gaze has also been associated with social anxiety symptoms (Gamer et al., 2011; Harbort et al., 2013; Jun et al., 2013; Schulze, Lobmaier, et al., 2013; Schulze, Renneberg, & Lobmaier, 2013).

Furthermore, individuals with autism spectrum conditions (ASC^1^) show differences in social cue integration when viewing images of isolated individuals (Ashwin et al., 2015; Mihalache et al., 2020); autistic observers focus more on body than head information (Ashwin et al., 2015) and utilise information from the eyes less than non-autistic individuals (Mihalache et al., 2020), when judging the direction of an individual’s gaze. These findings are potentially explained by their enhanced perception of features at the expense of global processing (Happé, 1999). Increased reliance on one cue, and aberrant integration of cues from the eyes, head, and body when judging gaze direction, could lead to inaccurate gaze perception, leading to difficulties in successfully identifying and responding to social interactions. However, the nature and extent of cue integration during perception of social interactions in autistic individuals are relatively unknown. Individual social cues can be perceived differently if we make judgements about them from a first-person (egocentric) perspective versus from a third-person perspective (Balsdon & Clifford, 2018). Relatedly, it is unclear whether autistic symptoms, which are typically associated with differences in social processing, modulate social cue integration across allocentric and egocentric frame(s) of reference (FoR).

It remains unknown how cue integration works when social interactions are viewed from third-person perspectives, and how allocentric and egocentric FoR influence judgements of dyadic interactions. Thus, in the first experiment, we sought to investigate whether observers integrate directional cues from the body with head orientation information when judging whether two people are interacting, using well-controlled, computer-generated stimuli that systematically vary in head and body orientation. Importantly, we occluded the visibility of the eye-region with dark sunglasses such that any judgements of interaction may be made based on information from the orientation of the head and body, rather than directly from the eye-region. Similar to Moors et al. (2015), this study examines how body orientation influences assumed gaze direction. In addition, we investigated whether cue integration is influenced by FoR (i.e., allocentric vs. egocentric), and whether autistic traits affect the nature of social cue integration during the perception of social interactions.

## Experiment 1

### Methods

#### Open science statement

The study was pre-registered on AsPredicted.org. In line with open science initiatives (Munafò et al., 2017), data and stimuli from this study are freely available online, and we report all data exclusions and measures in the study.

#### Participants

Participants were recruited via Amazon’s Mechanical Turk and were paid $7.00 for 30–45 min of their time. Studies investigating individual differences are likely to find small effect sizes (Schäfer & Schwarz, 2019); thus, to investigate the impact of autistic traits on interaction perception, a sample of *N* = 120 allows us to detect small effect sizes with 80% power.

As the study was conducted online, participant data were only included in the final data set if their total attention score was above 75% (attention checks are detailed in the “Procedure” section); data from a total of *N* = 131 participants were included in the final data set. However, after applying the exclusion criteria as detailed in the “Data Analysis” section, *N* = 118 participants remained in the analysis (*M*_age_ = 37.75, *SD* = 7.65, 60 females). All participants provided written informed consent, and the experiment was approved by the University of Reading, School of Psychology and Clinical Language Sciences Ethics Committee (ethical approval number: 2020-098-BC) and conducted in line with ethical guidelines presented in the 6th (2008) Declaration of Helsinki.

#### Stimuli

**Figure 2.**
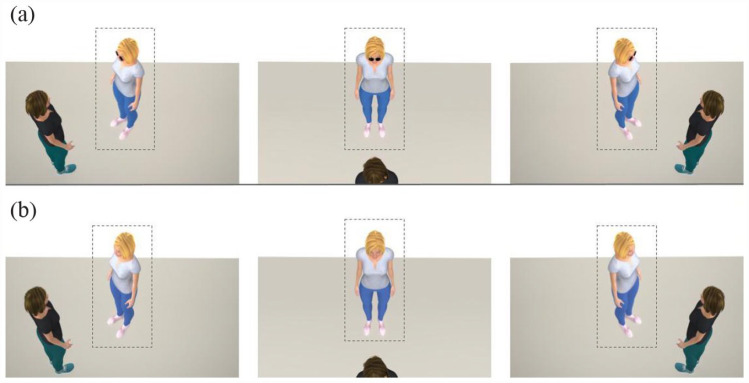
Examples of stimuli presented in (a) Experiment 1 and (b) Experiment 2. Stimuli containing dyads in neutral/interacting positions across Condition 1 (allocentric), Condition 2 (egocentric), and Condition 3 (allocentric). The head orientations of the moving avatar (outlined with a dashed rectangle for illustration purposes) varied from –30° to +30° in steps of 5°, and the body was turned –30°, 0°, or +30°.

Stimuli containing two female avatars presented within three different scenes/conditions were developed using Poser 12 software (Bondware, Inc.). Three scenes were developed to represent egocentric and allocentric FoR; Conditions 1 and 3 acted as proxies for an allocentric FoR (n.b. these conditions are identical but horizontally flipped), and Condition 2 acted as a proxy for an egocentric FoR (Figure 2a; see the online supplementary information 2 for further examples of stimuli).

Within each of the three FoR, the head and body orientation of one of the avatars remained static, while the other’s head and body orientation varied systematically. In Condition 1 (allocentric), the static avatar was positioned to the left of the screen with a head and body orientation of 125° relative to the observer, while the moving avatar was positioned centrally in the scene with a neutral head and body position of 305° relative to the observer (n.b. the neutral position of the moving avatar represents the veridical “interacting” response, as this is where both avatars directly face each other). The head orientation of the moving avatar ranged from –30° left to 30° right of the static avatar in steps of 5°, creating 13 unique head orientations. The body of the moving avatar was oriented –30° left, 30° right, or directly facing (0°) the static avatar. Although similar to Condition 1, the static avatar in Condition 3 (allocentric) was positioned to the right of the screen (215° relative to the observer; a horizontal flip of Condition 1). The static avatar in Condition 2 (egocentric) was positioned in the centre of the screen and turned 180° relative to the observer so that only the back of their head/body was visible, while the moving avatar’s neutral (or veridical interacting) position was directly facing the observer (0°). The camera position (X = 0°, Y = 4°, Z = 41°) was elevated such that both avatars would be visible across all three conditions.

#### Procedure

The experimental task was hosted on Gorilla Experiment Builder (www.gorilla.sc). Participants were restricted to completing the task from a laptop or desktop computer (64% used the Chrome browser, 5% used Firefox, 4% used Edge, and the browser type was not recorded for 27% of participants). Each trial began with a central fixation cross presented for 500 ms. A blank screen then appeared for 100 ms before a static image of a dyad (image size: 1085 × 822 pixels) was displayed at full resolution for 750 ms. After the presentation of the dyad, participants were asked to respond as to whether or not the dyad was interacting (two-alternative forced choice task; 2AFC). Participants used the “Y” and “N” letters on the keyboard to record “Yes” and “No” responses, respectively; the next trial started after participants made a response. Participants first completed nine practice trials to get acquainted with the task; the practice trials displayed only trials in which the answer to the question, “Are these two people interacting?” was clear (e.g., a head oriented –30° presented with a body oriented –30° should be a simple “No” response, and a head oriented 0° presented with a body oriented 0° should be a simple “Yes” response). Subsequently, with six repetitions of each combination of head, body, and FoR, participants completed a total of 702 trials across six blocks. Breaks could be taken in between blocks of trials. As the task was completed online, attention checks were presented randomly throughout to ensure participants were engaged with the task. To reduce the likelihood of submission from bots and random responding from participants, we included free-text responses to simple questions (e.g., “How many characters did you see on the last screen?” “What is the date today?” or “What is your age?”).

To measure self-reported autistic traits, at the end of the task participants completed the Autism Spectrum Quotient (AQ) questionnaire (Baron-Cohen et al., 2001) (*M* = 20.22, *SD* = 8.63), which also included two catch-questions to reduce the likelihood of participants responding randomly. Only participants who scored above 75% across all attention trials were included in the analyses.

#### Data analysis

Data from the 2AFC task were pre-processed in MATLAB (version R2015b) in the same way as described in Balsdon and Clifford (2018). The proportion of “interacting” responses at each head orientation was fit with the difference between two logistic functions (i.e., if participants had been asked to judge the pointing direction of the head of a looker, rather than judge whether a dyad is interacting, one logistic function would be fit to increasing leftward head responses made by the participant as the head of the looker rotates further left, and one would be fit to increasing rightward head responses as the head rotates further right). The peak of the “interacting” responses (or the head orientation at which the maximum of these functions occurred) was interpreted as the head orientation that maximally signals interaction in the dyad. If the body orientation had no influence on interaction perception, then the head orientation associated with the highest “interacting” responses should be identical between the leftward- and rightward-oriented bodies. We could therefore assess whether observers integrate information from the body with information from the head when perceiving interaction by computing an estimate of the influence of body orientation on interaction perception; this was calculated by finding the difference between the head orientation at which the peak of “interacting” responses was observed for the leftward- and the rightward-oriented bodies, and dividing this difference by two (we assume that cue integration is identical across hemifields; Balsdon & Clifford, 2018; Palmer et al., 2018). This represents the average extent to which body orientation shifts interaction perception away from that indicated by head orientation alone. If this value is equal to 0°, then body orientation has no influence on interaction perception. A value greater than 0° would suggest that the orientation of the body leads to interaction being perceived in the direction opposite the body (i.e., overshoot/repulsive effect), while a negative value would suggest that interaction is perceived in the same direction as the body (i.e., towing/attractive effect; Figure 1). All subsequent statistical analyses were performed on the measure of the influence of body orientation on interaction perception, henceforth referred to as *Body Influence*.

Before analysis, participant data were excluded if the peak of the proportion of interacting responses was outside the range of head orientations presented (i.e., greater than +30° or smaller than –30°) (Balsdon & Clifford, 2018). Inspection of data from excluded participants revealed that *N* = 5 participants responded only to the orientation of the body and not to the orientation of the head, while others appeared to respond randomly, failing to follow experimental instructions (*N* = 8).

Using the lmerTest package (Kuznetsova et al., 2017) in R (version 4.1.2; R Core Team, 2021), we fit linear mixed-effects models using restricted maximum-likelihood to investigate whether body influence values were predicted by FoR, autistic traits, and their interaction. Participants were entered as random effects, and age and gender were included as fixed-effect covariates (formula: Body Influence ~ FoR × Autistic Traits + Age + Gender + [1| Participant]). Autistic traits and age were median-centred and scaled. Data from the two allocentric conditions (Conditions 1 and 3) were collapsed together to compare body influence during interaction perception across allocentric and egocentric FoR (see the online supplementary information 1). Sixteen influential observations (4.5%) were excluded based on the criterion Cook’s D greater than 4 times the average Cook’s D (>0.25). Significance of fixed effects from the mixed-model was determined using Satterthwaite approximations of degrees of freedom using the lmerTest package, limiting Type 1 errors but maintaining power (Luke, 2017).

### Results and discussion

**Figure 3.**
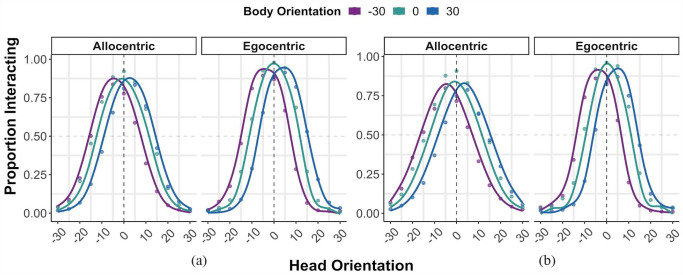
Responses to the 2AFC tasks; participants judged whether a dyad was interacting or not in (a) Experiment 1 and (b) Experiment 2. The vertical dashed lines intersecting 0° on the x-axis represent the head orientation at which the highest number of interacting responses should be observed if body information is not integrated with head information and has no influence on interaction perception. The peaks of the curves represent the head orientation at which participants mostly perceive the dyads to be interacting. The filled points show the actual proportion of responses, while the solid lines are calculated as the difference between two logistic functions, fitted by minimising the sum of squared error of the data points from the solid lines (data are averaged over all participants for illustration purposes). 2AFC: two-alternative forced choice task.

As shown in Figure 3a, the head orientation at which the peak of interacting responses was observed differed across body orientations. One-sample *t* tests showed that body influence was significantly different from zero across both allocentric, *t*(235) = 28.42, *p* < .001, and egocentric, *t*(117) = 22.90, *p* < .001, FoR. This suggests that body orientation is integrated with head orientation information when perceiving social interactions across different FoR. In addition, as illustrated in Figure 3a, participants perceived the moving avatar to be looking further away from the veridical direction of the head and in the direction opposite the body when the body was oriented to the left or to the right, demonstrating an overshoot effect; this was confirmed by positive body influence values in both allocentric (estimated marginal mean [EMM] = 3.54°, *SE* = 0.15, 95% confidence interval [CI] = [3.25, 3.83]) and egocentric (EMM = 4.08°, *SE* = 0.16, 95% CI = [3.76, 4.41]) FoR.

**Figure 4.**
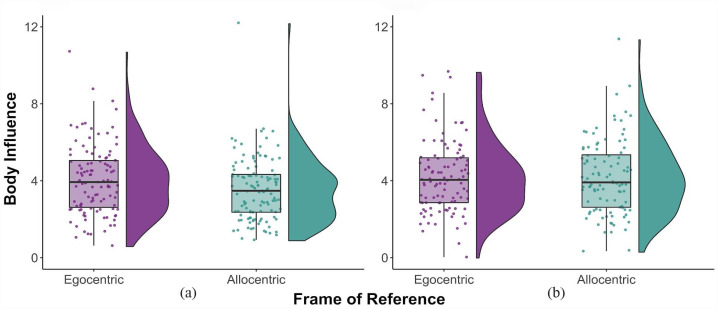
Body influence across FoR in (a) Experiment 1 and (b) Experiment 2. The coloured points show each participant’s body influence values, the boxplots represent the 25th and 75th percentiles, and the whiskers represent upper and lower values within 1.5*interquartile range. The “violins” show the distribution of the data, and their widths correspond to the probability density at each body influence value. FoR: frame(s) of reference.

The linear mixed-effects model (Table 1) revealed that the influence of the body on interaction perception was significantly predicted by FoR (Figure 4a); the influence of the body, which corresponded to an overshoot effect, was larger during the egocentric FoR compared with the allocentric FoR, β = 0.26, *SE* = 0.06, *t*(217.58) = 4.03, *p* < .001, 95% CI = [0.13, 0.39].

**Table 1.** Linear mixed-effects model summary for Experiment 1 and Experiment 2.

Predictors	Experiment 1				Experiment 2			
	β	CI	*T*	*p*	β	CI	*T*	*p*
Frame of reference (FoR)	0.26	[0.13, 0.39]	4.03	**<.001**	−0.00	[−0.14, 0.14]	−0.02	.981
Autistic traits (AQ)	0.11	[−0.17, 0.39]	0.77	.440	0.35	[0.01, 0.70]	2.03	**.044**
Age	−0.00	[−0.29, 0.28]	−0.03	.976	0.23	[−0.12, 0.58]	1.31	.192
Gender	0.27	[−0.01, 0.54]	1.90	.058	0.09	[−0.26, 0.43]	0.51	.613
FoR × AQ	−0.09	[−0.22, 0.03]	−1.46	.144	−0.13	[−0.27, 0.00]	−1.91	.057
Random effects								
σ^2^	1.21				1.27			
τ_00_	1.87 _PID_				2.64 _PID_			
ICC	0.61				0.68			
*N*	118 _PID_				104 _PID_			
Observations	338				297			
Marginal *R*^2^	.052				.044			
Conditional *R*^2^	.628				.690			

CI: confidence interval; ICC: intraclass correlation; PID: Participant ID. Bolded font indicates *p* values less than 0.05.

Experiment 1 sought to investigate whether observers integrate directional cues from the body and the head when judging whether two people are interacting while manipulating FoR and measuring participant-reported autistic traits. In line with previous studies investigating cue integration during the perception of isolated individuals (Moors et al., 2015), we found that participants integrated body information with head orientation information when perceiving social interactions. In addition, we replicated the overshoot effect observed in studies investigating eye and head integration (Moors et al., 2016) and head and body integration (Moors et al., 2015).

Furthermore, we found that observers integrated head and body cues differently across allocentric and egocentric FoR. Participants were more influenced by the body, corresponding to a stronger overshoot effect, in the egocentric compared with the allocentric FoR. It is possible that participants were weighting the directional cues differently depending on whether they were making egocentric (i.e., self-referential) compared with allocentric judgements. However, the increased salience of the body cue in the egocentric condition might be driving this difference. One possibility is that the eye-region of the interactant was less visible in the egocentric condition (Condition 2 in Figure 2) compared with the allocentric conditions. This relative lack of visibility could have resulted in a greater reliance on body directional cues for making the required judgement.

**Figure 5.**
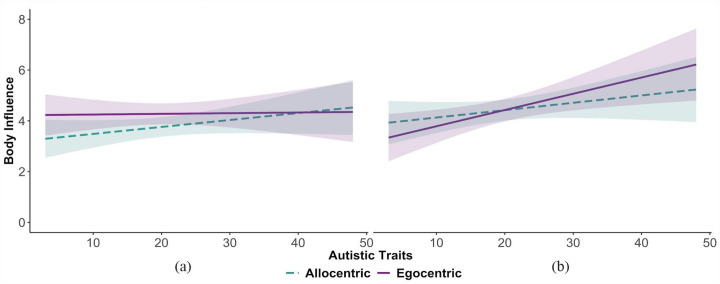
Body influence values in (a) Experiment 1 and (b) Experiment 2, across FoR and as a function of autistic traits.

We found no relationship between autistic traits and cue integration during interaction perception, nor an interaction between autistic traits and FoR in Experiment 1 (Figure 5a). Previous research has shown that autism is associated with differences in cue integration during gaze perception (Ashwin et al., 2015; Mihalache et al., 2020); autistic participants utilise information from the eyes less than non-autistic participants (Mihalache et al., 2020) and focus more on body information (Ashwin et al., 2015) when judging gaze direction. Indeed, diminished attention to others’ eyes is an early symptom of ASC (Jones & Klin, 2013). The gaze aversion hypothesis proposes that autistic individuals avoid looking at others’ eyes as they find direct eye-contact socially threatening (Hutt & Ounsted, 1966; Joseph et al., 2008; Kliemann et al., 2010; Kylliäinen & Hietanen, 2006). While interpreting the lack of evidence needs to be done with caution, a possible explanation for not finding an effect of autistic traits on cue integration during interaction perception in our study could be because observers were only required to integrate head and body information of dyads, as opposed to also having to integrate eye-region information. Consequently, it may be that individuals reporting more autistic traits show no differences in integrating head and body cues alone, but might show differences when eye-region information is visible. In light of the above, Experiment 2 sought to investigate whether autistic traits affect cue integration when observers judge whether two individuals are interacting, when their eye-regions, heads, and body information are visible.

## Experiment 2

### Methods

Methods were the same in Experiment 2 as Experiment 1, except for a change in stimuli as detailed below.

#### Participants

A total of 112 participants, who were distinct from the participants in Experiment 1, were recruited in the same manner as in Experiment 1. After applying the exclusion criteria as previously described, *N* = 104 participants remained in the analysis (*M*_age_ = 37.15, *SD* = 7.06, 48 females). All participants provided informed consent and the experiment was conducted in line with ethical guidelines presented in the 6th (2008) Declaration of Helsinki.

#### Stimuli

The stimuli in Experiment 2 remained the same as the stimuli presented in Experiment 1, with the crucial exception that the eye-region of the avatars was visible (Figure 2b). The eye direction was aligned with that of the head (i.e., the orientation of the eyes always moved congruently with the orientation of the head).

#### Procedure

Participants completed the same task as described in Experiment 1, except for the change in stimuli as detailed above. After completing the experimental task, participants completed the AQ questionnaire (*M* = 19.91, *SD* = 7.76).

#### Data analysis

Analysis was conducted in the same manner for Experiment 2 as detailed in Experiment 1. Following the same exclusion criteria after data processing but before data analysis, inspection of data from excluded participants revealed that *N* = 5 participants responded only to the orientation of the body and not to the orientation of the moving avatar’s head, while *N* = 3 appeared to respond randomly to the task.

As in Experiment 1, data from the two allocentric conditions were collapsed together to compare body influence during interaction perception across allocentric and egocentric FoR (see the online supplementary information 1). After fitting the data to the linear mixed-model, 15 influential observations (4.8%) were excluded based on the Cook’s D criterion greater than 4 times the average Cook’s D (>0.26).

### Results

As observed in Experiment 1, one-sample *t* tests showed that body influence was significantly different from zero across both allocentric, *t*(207) = 26.52, *p* < .001, and egocentric, *t*(103) = 21.53, *p* < .001, FoR (Figure 3b). This suggests that body orientation is integrated with head orientation information when perceiving social interactions across different FoR. In addition, as shown in Figure 3b, participants perceived the moving avatar to be looking further away from the veridical direction of the head when the body was oriented to the left or to the right, demonstrating an overshoot effect (i.e., interaction was perceived in the direction opposite the body orientation); this was confirmed by positive body influence values in both allocentric (EMM = 4.24°, *SE* = 0.18, 95% CI = [3.89, 4.60]) and egocentric (EMM = 4.24°, *SE* = 0.20, 95% CI = [3.85, 4.63]) FoR.

The results from the linear mixed-model (Table 1) showed no significant effect of FoR, β < –0.01, *SE* = 0.07, *t*(188.20) = –0.02, *p* = .981, 95% CI = [–0.14, 0.14] (Figure 4b). However, a significant effect of autistic traits was observed, β = 0.35, *SE* = 0.18, *t*(97.65) = 2.03, *p* = .046, 95% CI = [0.01, 0.70], and a marginally significant interaction between FoR and autistic traits, β = –0.13, *SE* = 0.07, *t*(187.58) = –1.91, *p* = .057, 95% CI = [–0.27, 0.01] (Figure 5b). Simple slopes analyses were performed on the marginal interaction effect. The slope of autistic traits was significantly different from zero in the egocentric FoR, β = 0.49, *SE* = 0.20, *t* = 2.48, *p* = .01, but not in the allocentric FoR, β = 0.22, *SE* = 0.18, *t* = 1.23, *p* = .22.

#### Exploratory analysis and results

While a significant relationship between autistic traits and body influence was found in Experiment 2, this relationship was not observed in Experiment 1. Conversely, a significant relationship between FoR and body influence was found in Experiment 1, but this was not shown in Experiment 2. Although two different samples of participants were tested across experiments, the only difference in experimental design is the eye-region visibility of the dyads. Thus, the findings were further explored by combining the two independent data sets from Experiment 1 and Experiment 2 (n.b. this exploratory analysis was not pre-registered) and fitting the data to a linear mixed-effects model using restricted maximum-likelihood (formula: Body Influence ~ FoR × Autistic Traits × Experiment + Age + Gender + [1| Participant ID]).

Twenty-nine influential observations (4.4%) were excluded based on the Cook’s D criterion greater than 4 times the average Cook’s D (>0.15). The model (Table 2) showed significant effects of FoR, β = –0.13, *SE* = 0.05, *t*(410.45) = –2.78, *p* = .006, 95% CI = [–0.23, –0.04], and autistic traits, β = 0.25, *SE* = 0.12, *t*(216.10) = 2.18, *p* = .031, 95% CI = [0.03, 0.48]; a significant two-way interaction between FoR and experiment, β = 0.11, *SE* = 0.05, *t*(410.59) = 2.38, *p* = .019, 95% CI = [0.02, 0.21]; and a three-way interaction between FoR, experiment, and autistic traits, β = –0.12, *SE* = 0.05, *t*(408.18) = –2.59, *p* = .010, 95% CI = [–0.22, –0.03] (Figure 5).

**Table 2.** Linear mixed-effects model summary for exploratory analysis.

Predictors	Exploratory analysis			
	β	CI	*T*	*p*
Frame of Reference (FoR)	−0.13	[−0.23, –0.04]	−2.78	**.006**
Autistic Traits (AQ)	0.25	[0.03, 0.48]	2.18	**.030**
Experiment (Exp)	0.22	[−0.01, 0.45]	1.89	.059
Age	0.09	[−0.14, 0.32]	0.76	.450
Gender	0.17	[−0.06, 0.39]	1.46	.144
FoR × AQ	−0.02	[−0.12, 0.07]	−0.44	.658
FoR × Exp	0.11	[0.02, 0.21]	2.36	**.019**
AQ × Exp	0.13	[−0.10, 0.36]	1.11	.266
FoR × AQ × Exp	−0.12	[−0.22, –0.03]	−2.59	**.010**
Random effects				
σ^2^	1.24			
τ_00 PID_	2.41			
ICC	0.66			
*N*_PID_	222			
Observations	637			
Marginal *R*^2^	.051			
Conditional *R*^2^	.679			

CI: confidence interval; ICC: intraclass correlation; PID: Participant ID. Bolded font indicates *p* values less than 0.05.

To investigate the significant two-way interaction between the categorical fixed effects, Tukey-adjusted pairwise comparisons were performed using the R package “emmeans” (Lenth et al., 2019). This showed that the influence of the body on interaction perception was larger when the eyes were visible in Experiment 2 in the allocentric FoR, compared with when the eyes were obscured in Experiment 1, β = 0.66, *SE* = 0.24, *t*(250) = 2.80, *p* = .028. In addition, the influence of the body on interaction perception was larger during the egocentric condition compared with the allocentric condition when the eyes were obscured in Experiment 1, β = –0.51, *SE* = 0.13, *t*(414) = –3.99, *p* = .001, and during the egocentric condition when the eyes were visible (Experiment 2) compared with the allocentric condition when the eyes were obscured (Experiment 1), β = 0.67, *SE* = 0.25, *t*(301) = 2.69, *p* = .038.

Simple slopes analyses to investigate the three-way interaction effect (Figure 5) showed that the slope of autistic traits was significantly different from zero in the egocentric FoR when the eye-region was visible in Experiment 2 (β = 0.53, SE = 0.20, t = 2.61, p = .01), but not when the eye-region was obscured in Experiment 1 (β = 0.02, SE = 0.17, t = 0.13, p = .90).

## General discussion

Experiment 2 sought to replicate the findings from Experiment 1 and further explore whether autistic traits affect cue integration during perception of social interactions when the eye-regions, heads, and bodies of dyads are visible. In line with Experiment 1, we found that body orientation is indeed integrated with head orientation when perceiving social interactions. In addition, we replicated the overshoot/repulsive effect of body orientation on interaction perception, such that perceived interaction is shifted away from body orientation when head and body cues are misaligned. This is consistent with previous findings that body orientation exerts a repulsive influence on head orientation (Moors et al., 2015), and head orientation exerts a repulsive influence on gaze direction (Moors et al., 2016; Otsuka et al., 2014, 2015).

As discussed in the introduction, an explanation for the overshoot effect was proposed by Anstis et al. (1969) and Otsuka et al. (2014). Namely, an overshoot effect is created when the visible amount of white sclera on either side of the iris changes when a person’s eyes are fixated while the head rotates. Information from the eye-region was not visible to observers in Experiment 1, and extracting detailed information from the eye-region would be difficult in Experiment 2. Furthermore, as the eyes were always aligned with the head such that any information extracted from these cues would be congruent with each other, and the visible amount of sclera did not change across head rotations, it is not possible for the overshoot effect observed in our experiments to be explained by a change in eye-region information. A recent study by Moors et al. (2015) observed that the overshoot effect of the body increased with increasing misalignment between head and body cues; the authors suggest that increased misalignment between head and body cues in a looker creates a strong directional spatial code, indicating that the person is shifting their attention; thus, observers might implicitly assume that gaze is not aligned with the head due to implied motion. Therefore, observers in our study might have assumed that the eyes of the avatar were not aligned with the head because the misaligned head and body cues imply that the moving avatar is shifting its attention. It would be interesting for a future study to investigate the overshoot effect using stimuli where information from the body, head, and eye-region are all clearly visible to observers and are manipulated independently, to disentangle each cue’s influence on the overshoot effect.

In contrast to Experiment 1, observers did not integrate head and body cues differently across allocentric and egocentric FoR in Experiment 2. Participants in Experiment 1 showed a stronger overshoot effect of the body during the egocentric compared with the allocentric FoR, whereas participants in Experiment 2 were influenced by the body to the same extent across FoR; this was confirmed by a significant interaction between experiment and FoR in the exploratory analysis (Table 2). Given that the eye-region, a salient directional cue, is not visible in Experiment 1, it is possible that the relative weightings of head, body, and eye-region information differ to their weightings in Experiment 2. Where there is increased uncertainty for the eye-region cue in Experiment 1, the relative weights attached to the eye-region and potentially the head orientation will be reduced, consequently increasing the weighting of the body cue and increasing the overshoot effect, particularly in the egocentric condition where the body cue is most salient. This is consistent with previous discussions by Perrett and colleagues (1992) and Otsuka and colleagues (2014), who assume that weights attached to each directional cue during gaze perception are not fixed, but vary according to the viewing conditions (Gamer & Hecht, 2007), context (Balsdon & Clifford, 2018), and the information available within the stimuli.

Unlike Experiment 1, a relationship between autistic traits and the influence of the body on interaction perception was observed in Experiment 2; participants with higher AQ scores had higher body influence values (i.e., exhibiting a stronger overshoot effect) than those with lower AQ scores. The marginal interaction between autistic traits and FoR in Experiment 2 (Table 1) demonstrated that observers with high AQ scores were influenced more by the body in the egocentric than in the allocentric condition; this effect was supported by a significant three-way interaction between autistic traits, FoR, and experiment in the exploratory analysis (Table 2). Notably, the only difference between Experiments 1 and 2 was the visibility of the eye-regions of the dyads; thus, it is possible that the discrepancies in findings across experiments are due to whether or not the eye-region is visible to observers. As previously discussed, autistic individuals utilise eye information less than non-autistic participants when making judgements about gaze (Mihalache et al., 2020) and focus more on body information than head and eye information in a spatial cueing paradigm (Ashwin et al., 2015). In addition, the gaze aversion hypothesis (Hutt & Ounsted, 1966; Joseph et al., 2008; Kliemann et al., 2010; Kylliäinen & Hietanen, 2006) suggests that autistic individuals actively avoid looking towards the eye-region because they find the eyes aversive. Accordingly, individuals reporting more autistic traits in Experiment 2 might assign lower weightings to eye-region and head orientation cues of dyads when perceiving interactions, thus becoming more susceptible to the repulsive effect of the body.

However, the effect of AQ was only observed in the egocentric FoR; it is possible that participants with more autistic traits find a frontal view of the eyes more aversive than a side view of the eyes, leading to reduced attention to the eye and head cues in this condition. Relatedly, it could be argued that the effect of AQ is observed only when participants engage in self-referential judgements. Indeed, patients with schizophrenia (Hooker & Park, 2005; Rosse et al., 1994; Tso et al., 2012) and social anxiety (Gamer et al., 2011; Harbort et al., 2013; Jun et al., 2013; Schulze, Lobmaier, et al., 2013; Schulze, Renneberg, & Lobmaier, 2013) show differences in self-referential gaze perception. In addition, Balsdon and Clifford (2018) observed that participants weighted head and eye cues differently depending on whether they were making directional (i.e., allocentric) or self-referential (i.e., egocentric) judgements. It is important to note that the stimuli presented in our study acted only as proxies for egocentric and allocentric FoR; we acknowledge that the ecological validity of these stimuli is limited due to the unnatural positioning of the camera in both conditions. It would be interesting for future studies to compare the influence of the body on interaction perception in tasks more directly comparing directional versus self-referential judgements.

In interpreting our findings, it is important to consider the limitations. First, both experiments discussed in this article were conducted completely online during the covid-19 pandemic. Although there has recently been a surge in research conducted online, and carefully designed online experiments can offer reliable data that are indistinguishable from data collected in the lab (Crump et al., 2013; Germine et al., 2012), we acknowledge limitations associated with online testing, especially the lack of control of a participant’s environment, including their viewing distance and angle (though see Heer & Bostock, 2010 and Liu & Heer, 2018). Nevertheless, it is promising that we demonstrated that participants integrate body information with head information when perceiving social interactions, and replicated the previously found overshoot effect, across two large-sampled experiments. Second, although we report a relationship between autistic traits and interaction perception, we do not know whether this relationship extends to participants with clinical ASC diagnoses. It would be valuable for future studies to attempt to replicate our findings in a lab-setting among a sample with a clinical diagnosis of ASC. Third, in favour of experimental control, static displays of social interactions were presented to participants; although this allowed for easier presentation of various combinations of head and body angles, we acknowledge that real-world perception of social interaction is much more dynamic and unpredictable, and our results provide only a first approximation of cue integration during perception of social interactions in the real world. Indeed, dynamic stimuli might convey more information about the intentions of the dyads and might thus lead to different integration of eye, head, and body information. Relatedly, although the eye-region of the dyads was not occluded in Experiment 2, observers would be limited in their ability to extract detailed information about the direction in which their eyes were pointing. Thus, any effects of eye-region visibility observed in our study might be due to observers implicitly assuming where the eyes of the dyads were looking.

The results of this study indicate that body information is integrated with head information when perceiving social interactions such that perceived interaction is shifted away from body orientation when head and body cues are misaligned. In addition, our findings suggest that autistic traits and FoR affect cue integration during interaction perception, but that these effects are dependent on the visibility of the eye-region. The results provide crucial first insights into how directional cues are integrated during interaction perception across different contexts, as well as an important contribution to our understanding of social cue integration in individuals with and without autism.

## Supplemental Material

sj-docx-1-qjp-10.1177_17470218231181001 – Supplemental material for The integration of head and body cues during the perception of social interactions Supplemental material, sj-docx-1-qjp-10.1177_17470218231181001 for The integration of head and body cues during the perception of social interactions by Elin H Williams and Bhismadev Chakrabarti in Quarterly Journal of Experimental Psychology

## Notes

1. We recognise the diversity of views on terminology within the autism community. To reflect this diversity of views, we use the phrase “individuals with Autism Spectrum Conditions” interchangeably with “autistic individuals.”
