# Supplementary material for: The integration of head and body cues during the perception of social interactions
Source: Q J Exp Psychol (Hove). 2023 Jun 22;77(4):776–88. doi: 10.1177/17470218231181001 (PMC10960325; doi:10.1177/17470218231181001)

Supplementary Material for:

**The Integration of Head and Body Cues during the Perception of Social Interactions**

*Elin H. Williams^1*^ & Bhismadev Chakrabarti^1,2,3^*

^1^Centre for Autism, School of Psychology and Clinical Language Sciences, University of Reading, Reading, UK

^2^India Autism Centre, Kolkata, India

^3^Department of Psychology, Ashoka University, India

* to whom correspondence should be addressed

Elin H. Williams

School of Psychology and Clinical Language Sciences,

University of Reading,

Reading,

RG6 6DZ

[e.h.williams@reading.ac.uk](mailto:e.h.williams@reading.ac.uk)

**Supplementary Information 1**

In order to investigate whether allocentric and egocentric frames of reference influence cue integration during interaction differently, data from the two allocentric conditions (Conditions 1 and 3) were collapsed together. Paired samples t-tests confirmed that Condition 1 and Condition 3 were not statistically different from each other in neither Experiment 1 nor 2 (Table S1).

**Table S1. Results of paired t-tests comparing means from Conditions 1, 2, and 3.**

|  | **Mean difference** | **t** | **df** | **95% CI** | ***p*** |
| --- | --- | --- | --- | --- | --- |
| ***Experiment 1*** |  |  |  |  |  |
| Condition 1 v Condition 2 | -0.67 | -4.08 | 117 | -1.00 - -0.35 | **<0.001** |
| Condition 1 v Condition 3 | -0.20 | -1.39 | 117 | -0.49 - 0.08 | 0.17 |
| Condition 2 v Condition 3 | 0.47 | 2.57 | 117 | 0.11 - 0.84 | **0.01** |
| ***Experiment 2*** |  |  |  |  |  |
| Condition 1 v Condition 2 | -0.02 | -0.07 | 103 | -0.45 - 0.41 | 0.94 |
| Condition 1 v Condition 3 | -0.13 | -0.69 | 103 | -0.50 - 0.24 | 0.49 |
| Condition 2 v Condition 3 | -0.11 | -0.65 | 103 | -0.46 - 0.23 | 0.52 |

*Condition 1 = allocentric, Condition 2 = egocentric, Condition 3 = allocentric.*

**Supplementary Information 2**

*Supplementary Figure 1.* Additional examples of stimuli presented in (A) Experiment 1 and (B) Experiment 2. The head orientation of the moving avatar (outlined with a dashed rectangle for illustration purposes) is set at +20° in the examples shown above in (A) and -20° in (B). The body orientation of the moving avatar is set at -30° in the examples shown above in (A) and +30° in (B).
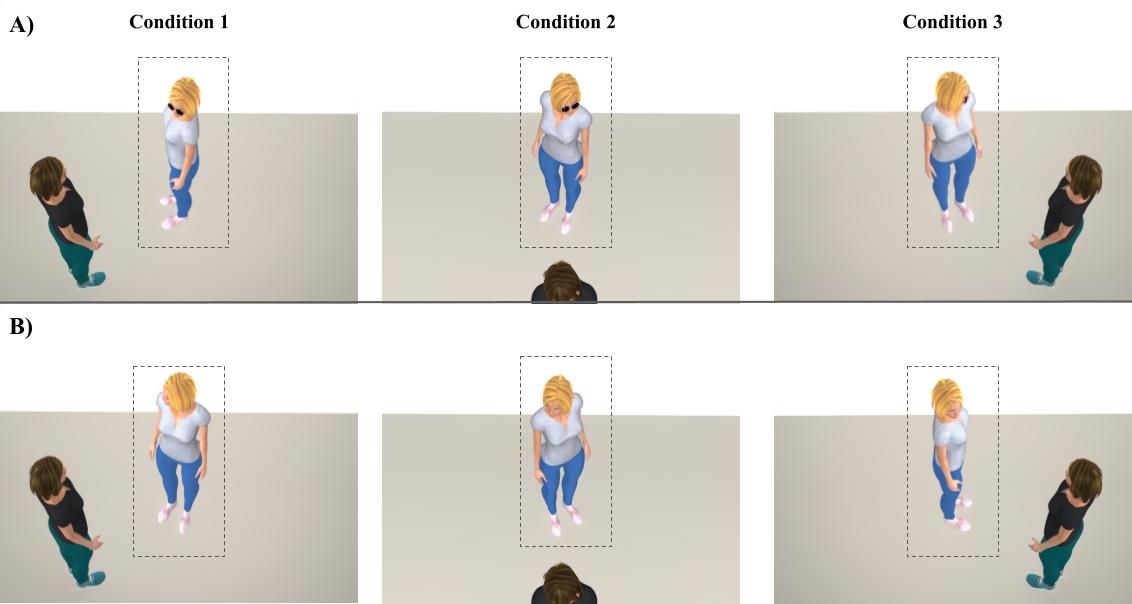

Supplement: sj-docx-1-qjp-10.1177_17470218231181001 – Supplemental material for The integration of head and body cues during the perception of social interactions [file sj-docx-1-qjp-10.1177_17470218231181001.docx]
